# The role of smart polymeric biomaterials in bone regeneration: a review

**DOI:** 10.3389/fbioe.2023.1240861

**Published:** 2023-08-17

**Authors:** Yanghui Xing, Linhui Qiu, Danqing Liu, Sihan Dai, Chia-Lin Sheu

**Affiliations:** Department of Biomedical Engineering, Shantou University, Shantou, China

**Keywords:** smart biomaterials, bone regeneration, stimuli, polymer, osteogenic

## Abstract

Addressing critical bone defects necessitates innovative solutions beyond traditional methods, which are constrained by issues such as immune rejection and donor scarcity. Smart polymeric biomaterials that respond to external stimuli have emerged as a promising alternative, fostering endogenous bone regeneration. Light-responsive polymers, employed in 3D-printed scaffolds and photothermal therapies, enhance antibacterial efficiency and bone repair. Thermo-responsive biomaterials show promise in controlled bioactive agent release, stimulating osteocyte differentiation and bone regeneration. Further, the integration of conductive elements into polymers improves electrical signal transmission, influencing cellular behavior positively. Innovations include advanced 3D-printed poly (l-lactic acid) scaffolds, polyurethane foam scaffolds promoting cell differentiation, and responsive polymeric biomaterials for osteogenic and antibacterial drug delivery. Other developments focus on enzyme-responsive and redox-responsive polymers, which offer potential for bone regeneration and combat infection. Biomaterials responsive to mechanical, magnetic, and acoustic stimuli also show potential in bone regeneration, including mechanically-responsive polymers, magnetic-responsive biomaterials with superparamagnetic iron oxide nanoparticles, and acoustic-responsive biomaterials. In conclusion, smart biopolymers are reshaping scaffold design and bone regeneration strategies. However, understanding their advantages and limitations is vital, indicating the need for continued exploratory research.

## 1 Introduction

Human bone structure, vital for mobility, structural support, and organ protection, has remarkable self-healing abilities (Stevens, 2008). However, critical-sized bone defects (CSD), resulted from tumor, trauma, infection, or other severe bone damages, is challenging due to problems such as immune rejection and donor shortage (Amini et al., 2012; Agarwal and García, 2015). An ideal bone scaffold for CSD should mimic the natural bone composition of collagen, hydroxyapatite, and cells. Additionally, the scaffold should eventually degrade, after serving its function (Battafarano et al., 2021). For this reason, polymeric materials have been extensively studied for bone repair, offering innovative properties for bone implant optimization, bone tissue engineering and therapeutic agent delivery (Laurencin and Khan, 2012; Ogueri et al., 2019; Filippi et al., 2020; Khan et al., 2022a). In principle, the material should emulate the properties of the surrounding tissue, be it rigidity for bone or pliability for softer tissues. Such properties are also influenced by the cellular requirements for porosity (Kohane and Langer, 2008).

Biomaterial factors such as biocompatibility, mechanical properties, and surface properties affect cell attachment, osteointegration, and osteogenesis (Ogle, 2015). Regeneration efficacy can be enhanced by delivering bioactive agents that regulate bone metabolic signaling pathways and new bone formation (Wei et al., 2022). Bone Tissue Engineering (BTE), a multidisciplinary field with decades of accumulated data, holds promise for addressing bone defects. BTE employs cells, growth factors, dynamic stresses, and biomaterials to fabricate bespoke bioactive scaffolds - including metals, ceramics, or polymers - to enhance bone repair (Wang et al., 2021).

Smart polymeric biomaterials are instrumental in controlled drug delivery systems, detecting stimuli and releasing bioactive agents accordingly (Wei et al., 2017; Montoya et al., 2021). Their function relies on stimuli-sensitive moieties that, when exposed to stimuli, undergo changes triggering drug release (Bustamante-Torres et al., 2021; Zhang et al., 2021). Stimuli-responsive biopolymers have recently gained attention as valuable graft materials. External physical triggers or certain pathological microenvironments can alter these materials’ configuration, influencing cell destiny and bolstering bone tissue therapy and regeneration (Sobol et al., 2011; Lavanya et al., 2020; Cerqueni et al., 2021; Sivakumar et al., 2022; Heng et al., 2023). This mini-review will delve into the major types of smart polymers used in bone regeneration, outlining their functions, advantages, and limitations.

## 2 Light responsive polymeric biomaterials for bone regeneration

Significant research underscores the utility of light-responsive polymers in precision drug delivery, boasting excellent control over spatial and temporal parameters and intensity, relevant in various medical conditions (Municoy et al., 2020; Pokharel and Park, 2022). In bone regeneration research, such materials can function as multifunctional scaffolds supporting bone repair or as drug delivery systems targeting antibacterial and osteogenic needs (Tomatsu et al., 2011).

**FIGURE 1:**
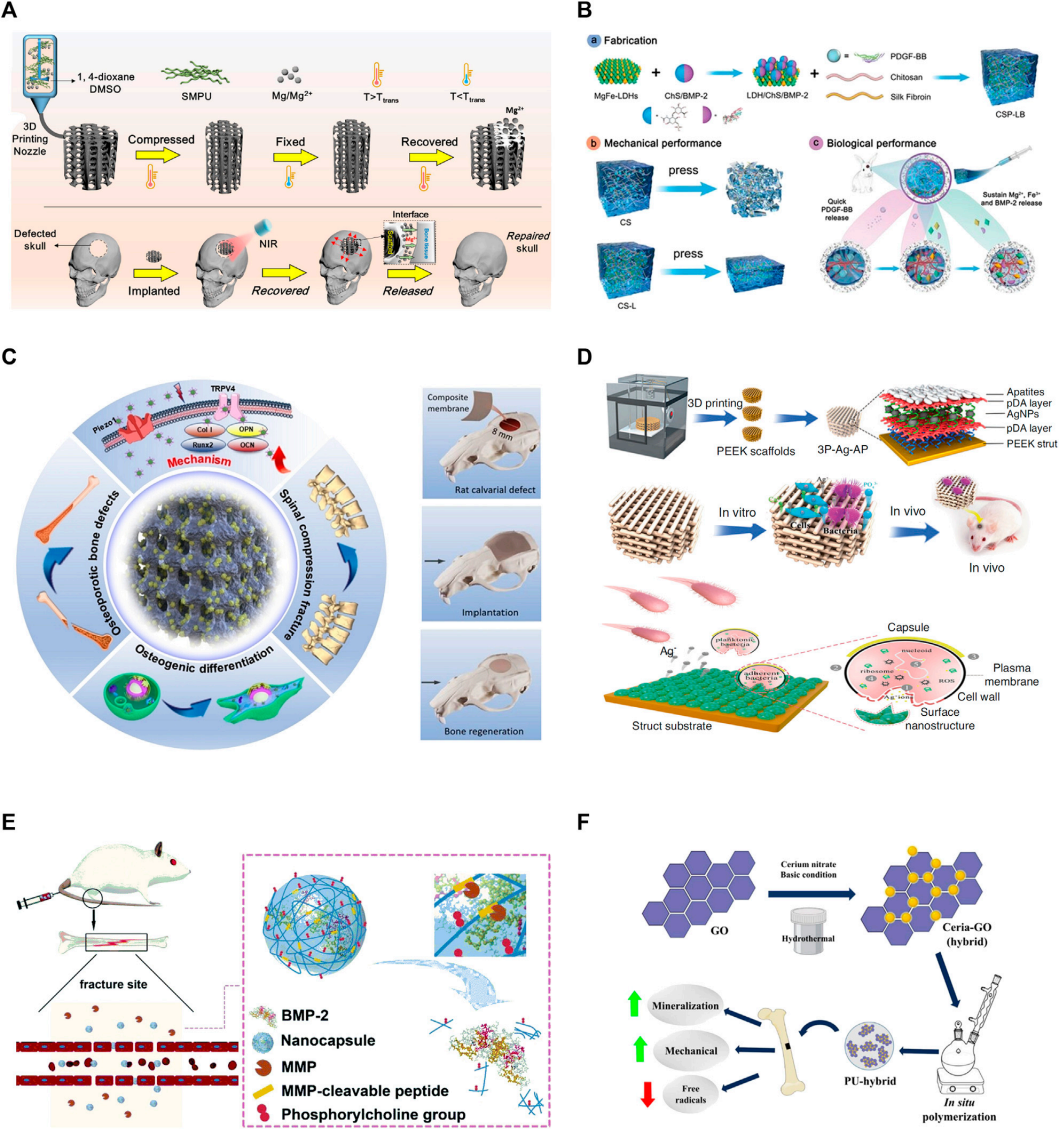
Representative mechanisms of smart polymeric biomaterials for bone regeneration. **(A)** Light-responsive polymers: NIR-induced tight contact of SMPU scaffold with bone structure and Mg^+^ release (Zhang et al., 2022). **(B)** Thermo-responsive polymers: Schematic illustration of the fabrication of CSP‐LB hydrogel, enhanced mechanical and biological properties (Lv et al., 2023). **(C)** Electrically-responsive polymers: Porous poly (vinylidene fluoride-co-hexafluoropropylene) (PVDF-HFP) and calcium phosphate (CaP) coatings created the scaffold (left); Barium titanate (BTO)/polyvinylidene fluoride–trifluoroethylene (P(VDF‐TrFE)) accelerate bone regeneration (right) (Ma Z. et al., 2023; Bai et al., 2023). **(D)** Enzyme-responsive polymers: MMP-induced BMP-2 release for fracture healing (Deng et al., 2020). **(E)** pH-responsive polymers: Bacteria-induced acid environments triggering drug release for anti-infection and osteogenesis (Qi et al., 2019). **(F)** Redox-responsive polymers: Ceria-Go-enhance radical-scavenging activity (Nilawar and Chatterjee, 2022).

One study reported a 3D-printed scaffold made of shape-memory polyurethane (SMPU) and magnesium (Mg) for bone repair. The implanted scaffold can form a tight contact within bone structure by changing its shape between original conformation and compressed conformation, which was controlled by near-infrared (NIR) irradiation-induced photothermal effects. The scaffold demonstrated significant osteo-promotive functions with *in vitro* and *in vivo* studies as shown in Figure 1A (Zhang et al., 2022). Photothermal therapy can also be used directly to bone defects, because heat at around 40°C–43°C can enhance proliferation and osteoblastic differentiation of mesenchymal stem cells (MSCs) (Liao et al., 2021). Tong et al. designed a biodegradable bone implant with black phosphorous (BP) nanosheets incorporated in poly (lactic-co-glycolic acid) (PLGA). After exposed to low intensity and periodic NIR, the implant significantly enhanced expressions of heat shock proteins, and increased osteogenesis in both cell and animal models (Tong et al., 2019). Furthermore, Zeng et al. developed a novel polydopamine-IR820-daptomycin coating for titanium bone implant. Under NIR irradiation, the composite had anti-bacterial efficiency. Additionally, the coating changed surface properties of implants, resulting in better contact with bones. The coating also significantly increased proliferation and osteogenic differentiation of bone marrow stem cells (Zeng et al., 2020).

Inflammatory response and low osteogenesis are two major issues that hinder bone regeneration. Kuang et al. developed a photo-responsive multicomponent hydrogel drug delivery system, which combined continuous drug release and NIR-controlled pulsatile drug release mechanisms together. In an osteoporosis animal model, the system can maintain parathyroid hormone (PTH) concentration in bone structure in a relatively stable manner, and thus promoted bone regeneration by achieving optimized osteoblast to osteoclast ratios (Kuang et al., 2021). In another study, osteo-inductive bone morphogenetic protein 2 (BMP-2) was attached to polydopamine-coated Mg-Ca carbonate microspheres which was incorporated into aspirin-containing hydroxybutyl chitosan (CS) hydrogel. The composite materials can release aspirin at early stage for anti-inflammatory effects, and then release BMP-2 to promote osteogenesis. Animal studies showed the presented composite promoted new bone formation (Wan et al., 2022). Furthermore, Wang et al. designed a NIR-triggered system with SrCl_2_-black phosphorous (BP)@PLGA microspheres, which can achieve on-demand release of Sr ions. After implanted into rat bone defects, the microspheres exhibit great biocompatibility and bone regeneration potential (Wang et al., 2018).

## 3 Thermo-responsive polymeric biomaterials for bone regeneration

Thermo-responsive biomaterials have piqued extensive interest, driven by their capacity for temperature-modulated bioactive agent release. Injectable systems enhance safety by transitioning phases without reliance on cross-linking agents, thereby avoiding denaturation. Moreover, the dynamic encapsulation process ensures therapeutic agents within the biomaterial. The rapid shift from a sol to a gel state at physiologic temperature eliminates the risk of premature burst release, optimizing the control of release kinetics (Duan et al., 2020).

Hydrogels’ therapeutic applications can be hampered by mechanical inadequacies and shrinkage during cell culture. To overcome this, Zhu et al. (2023) devised a thermo/photo dual-sensitive hydrogel, through physical and chemical cross-linking techniques, a thermo/photo dual-sensitive hydrogel was synthesized from methacrylated hydroxybutyl chitosan (MHBC) and chitin whisker (CHW). This M/C hydrogel exhibits a distinctive lamellar internal structure, and its mechanical properties and cellular compatibility can be tailored by modulating the M/C ratio.

A thermo-gel consisting of poly (ε-caprolactone-co-D,L-lactide)-poly (ethyleneglycol)-poly (ε-caprolactone-co-D,L-lactide) (PCLA-PEG-PCLA), simvastatin (SIM), strontium hydrogen phosphate (SrHPO_4_)/beta-tricalcium phosphate (beta-TCP) showcased superior osteocyte differentiation, facilitating bone tissue repair (Bian et al., 2023). Addressing periprosthetic wear debris-induced aseptic loosening, Lei et al. (2022) developed a thermosensitive PLGA-b-PEG-b-PLGA hydrogel. Infused with the TNF-alpha antagonist etanercept (ETN), the hydrogel mitigates debris-induced osteolysis through sustained ETN release, thereby reducing aseptic inflammation. For anterior cruciate ligament (ACL) repair, a thermos-responsive BP-FHE BP, primarily F127, oxidized hyaluronic acid (OHA), poly-epsilon-L-lysine (epsilon-EPL)) hydrogel was proven to promote mineralization, skin, and bone regeneration while reducing cytotoxicity, optimizing ACLR clinical use and recovery (Cho et al., 2023). Sui et al. (2023) L-PRF-based chitosan (CS)-hydroxyapatite (HAP) composite scaffold provides mechanical stability, sustained release, and enhanced cytotoxicity for bone regeneration. Another thermo-responsive hydrogel, chitosan/silk fibroin with platelet-derived MgFe-layered growth factor-BB (CSP-LB), incorporated with dual growth factors, exhibited improved angiogenesis, osteogenesis, bone regeneration, and mineral density compared to its CS counterpart, thanks to sequential growth factor release and sustained bioactive Mg^2+^/Fe^3+^ ion release as shown in Figure 1B (Khan et al., 2022b; Lv et al., 2023).

## 4 Electrically-responsive polymeric biomaterials for bone regeneration

The impact of electrical currents on bone formation is well-documented (Bassett et al., 1964), fostering the incorporation of conductive elements such as carbon nanofibers (Stout et al., 2012) and gold nanowires (Dvir et al., 2011) into conducting polymers. These polymers, soluble in organic solvents, can be blended with other polymers and processed into porous scaffolds, for instance, via electrospinning. Such uniformity enhances electrical signal transmission across the composite, influencing the behavior of all included cells.

Substantial strides have been made in scaffold designs for bone tissue engineering. For instance, a 3D-printed poly (l-lactic acid) (PLLA) scaffold was designed, featuring a fiber diameter of 150 μm and an osteogenic pore size of 450 μm, both crucial for bone growth. The design parameters were set to achieve the desired scaffold size. With impressive cytocompatibility, elasticity resembling that of trabecular bone, and inherent piezoelectric properties encouraging the adhesion of fibrinogen-coated osteoblast-like cells, these scaffolds exhibit significant promise (Karanth et al., 2023). Other notable developments include a 3D polyurethane foam (PUF) scaffold coated with piezoelectric PVDF-HFP and mineralized calcium phosphate (CaP), which stimulated osteogenic cell differentiation and *in vivo* ectopic bone formation due to its components’ synergistic effects (Ma et al., 2023a). BaTiO_3_ nanofibers (BTNF) integrated into a poly (vinylidene fluoridetrifluoroethylene) (P(VDF-TrFE)) matrix created an anisotropic surface potential, bolstering mechanotransduction, *in vitro* osteogenesis, and *in vivo* bone regeneration as shown in Figure 1C (Bai et al., 2023). Wang et al. (2023) suggested a composite scaffold consisting of piezoelectric Whitlockite (WH) and polycaprolactone (PCL) that fostered neurovascularized bone tissue regeneration through sustained Mg^2+^ release. A bifunctional composite formed by incorporating activated carbon nanotubes (ACNTs) into a polymethyl methacrylate (PMMA) matrix improved cell survival under electrical and magnetic stimuli (Li et al., 2022). A SiO_2_/PDMS composite electroactive membrane with embedded silicon dioxide electrets enhanced osteogenic differentiation and bone regrowth (Qiao et al., 2022). A pioneering approach introduced porous polymeric Fe_3_O_4_/GO scaffolds developed using cellulose and a co-dispersed nanosystem, exhibiting enhanced mechanical strength and antibacterial activities, as well as increased viability and proliferation of pre-osteoblast cell lines (Khan et al., 2022c). Lastly, a poly (l-lactic acid)-block-aniline pentamer (PLA-AP) and poly (lactic-co-glycolic acid)/hydroxyapatite (PLGA/HA-based) electroactive tissue engineering scaffold, loaded with the pSTAR-hBMP-4 plasmid, improved osteogenesis differentiation and bone healing, underlining the potential applications of multi-functional materials in bone tissue engineering (Cui et al., 2020).

## 5 pH-responsive polymeric biomaterials for bone regeneration

Osteoporosis, a metabolic bone disorder, arises from excessive osteoclast activity which breaks down bone structure via the secretion of acid and proteinases. Consequently, the pH of osteoporotic bones is lower than that of healthy ones (Blair, 1998). Bacterial infection, another major impediment to bone regeneration, can also lead to an acidic microenvironment around infection sites. Accordingly, pH-responsive polymeric biomaterials are predominantly used for the delivery of osteogenic and antibacterial drugs.

Deng et al. (2020) designed a dual-layer polydopamine coating for bone implants, incorporating silver nanoparticles (NPs) in the first layer and apatite in the second (Figure 1D). In response to bacterial infection, the coating releases Ag^+^, Ca^2+^, and PO_4_^3-^ ions. 3D-printed polyetheretherketone scaffolds modified with this coating demonstrated superior antibacterial and osteogenic properties *in vitro*, and promoted bone ingrowth and osseointegration *in vivo* in an infected bone defect. Another study introduced a drug release system composed of Poly [2-(dimethylamino) ethyl methacrylate] (PDMAEMA), chitosan, and a minocycline drug reservoir. As bacteria induce a pH reduction around the system, the pH-responsive PDMAEMA hydrogel propels the drug from the reservoir on-demand for bacterial inhibition with remarkable efficacy (Chen et al., 2023).

Synergistic effects of BMP-2 and dexamethasone (Dex) are critical for osteoblastic differentiation and bone regeneration. Gan et al. engineered a pH-sensitive, chitosan-functionalized mesoporous silica nanoparticle (chi-MSN). The design involves covalently attaching BMP-2 to chitosan and encapsulating smaller Dex molecules within the mesopores. Once delivered into cells, a lower pH triggers the release of Dex following the initial release of BMP-2. This system resulted in a substantial increase in osteoblastic differentiation and new bone formation *in vivo* over a period of 4 weeks (Gan et al., 2015). Finally, George et al. developed an injectable Oligo [poly (ethylene glycol) fumarate]-dopamine (OPF-DOPA) hydrogel that forms crosslinks under low pH conditions, subsequently increasing its stiffness and slowing its degradation rate. Notably, the hydrogel adheres to bone structures, preventing displacement of bone implants (George et al., 2022).

## 6 Enzyme-responsive polymeric biomaterials for bone regeneration

Enzymes are integral to bone growth and remodeling, modulating various signaling pathways within bone tissue such as cell proliferation, adhesion, and osteogenesis. Within the context of bone regeneration, native enzymes present in bone tissue, like matrix metalloproteinases (MMPs), can initiate specific reactions crucial for drug delivery, diagnostics, and tissue repair. Enzyme-responsive polymers have great biocompatibility, selectivity, and efficiency, and have excellent potential for bone regeneration.

Materials responsive to enzymes have also been employed to counteract bone infections. Polyglutamic acid (PG) is a homogeneous polymer featuring amide crosslinkers cleavable by the V8 enzyme, which is secreted by *Staphylococcus aureus*. Ding et al. (2020) encapsulated AgNPs into Mesoporous silica nanoparticles (MSNs), which were then enveloped by PG and polyallylamine hydrochloride (PAH) layers using a layer-by-layer technique. These nanoparticles were ultimately placed onto a polydopamine-coated surface as a titanium bone implant coating. The modified implants exhibited exceptional antimicrobial effects and significantly enhanced new bone formation in a bacteria-infected rat model. In a similar study for periodontal treatment, Alkaline Phosphatase (ALP) -responsive polyphosphoester and minocycline hydrochloride (PPEM) was incorporated into a chitosan membrane, and the effects were evaluated in cell and animal models. The results confirmed release of antibiotic and osteogenic drugs from PPEM membrane and their effects (Li et al., 2019).

In a recent study, researchers engineered Matrix metalloproteinases (MMP)-responsive nanocapsules to deliver bone BMP-2 for bone fracture healing. These nanocapsules, formed via *in situ* 2-(methacryloyloxy) ethyl phosphorylcholine (MPC) polymerization, incorporated the isacryloylated VPLGVRTK peptide as MMP cleavable crosslinkers on the BMP-2 surface, maintaining the functionality of BMP-2 throughout the process. The nanocapsules were delivered to fracture site via malformed blood vessels and accumulated there. Once MMPs disrupted the capsule, BMP-2 was released, facilitating bone regeneration as demonstrated with *in vivo* studies as shown in Figure 1E (Qi et al., 2019). Various other enzymes, including tyrosinase, lysozyme, horseradish peroxidase, transglutaminase (TG), and alkaline phosphatase (AP), have been examined for their potential to induce beneficial reactions for bone regeneration (Yuan et al., 2018; Sood et al., 2022).

## 7 Redox-responsive polymeric biomaterials for bone regeneration

Redox signaling pathways, predicated on electron transfer and free radicals, underpin mammalian bone formation and regeneration, especially balancing reactive oxygen species (ROS) (Zhang et al., 2023). Recent discoveries underline the post-fracture influence of redox on cellular responses.

Well-known for their role in redox modulation, ceria nanoparticles have been incorporated into a polyurethane matrix alongside graphene, creating a multifunctional biomaterial. Ceria-graphene oxide hybrid nanoparticles were synthesized through a hydrothermal process that started with the sonication of graphene oxide in distilled water. Following this, cerium nitrate hexahydrate was incorporated into the mixture and stirred magnetically. The pH was elevated to 10 through the addition of an ammonia hydroxide solution, and stirring was continued. The composite was then placed into a Teflon-lined stainless steel hydrothermal reactor and kept in an oven. Particles were dried in a hot air oven. Ceria nanoparticles and reduced graphene oxide sheets were similarly synthesized, albeit without adding graphene oxide and the cerium precursor, respectively. The end products displayed enhanced properties, specifically in terms of radical scavenging and osteogenesis (Nilawar et al., 2023). Ceria-graphene oxide hybrid nanoparticles were synthesized via a hydrothermal process and demonstrated heightened radical-scavenging and osteogenic properties. The bioactivity of 3D-printed, porous PLA scaffolds can be augmented by ceria, fostering osteogenesis enhancement and antimicrobial properties as shown in Figure 1F (Nilawar and Chatterjee, 2022). Further, nanoceria-cellulose-gelatin scaffolds (CG-NCs) have been crafted to combat ROS-induced oxidative stress inhibiting bone repair, boasting superior mechanical properties, bio-mineralization capabilities, and promoting cell proliferation and differentiation (Singh et al., 2023).

Gelatin methacrylate (GelMA) hydrogels, enhanced with magnesium-seamed C-propylpyrogallol[4]arene (PgC (3) Mg), offering dual-release of bioactive Mg^2+^ and antioxidants, boosting bioactivity and resilience to oxidative stress. The modified hydrogels decreased intracellular ROS levels and improved bone repair in severe cranial defects (Tan et al., 2022). Moreover, scaffold combining radially aligned mineralized collagen (RA-MC) fibers and nanosilicon (nSi) exhibited osteoconductivity and osteoinductivity, guiding reparative cells and reducing inflammation, thus showing promise for major bone defect repair (Mac et al., 2022). Additionally, sodium alginate hydrogel, embedded with calcium peroxide nanoparticles and vitamin C, has shown promising results in alleviating bone defect hypoxia and promoting bone healing under hypoxic conditions (Zhao et al., 2021). YQ Chen’s research focuses on enhancing the biocompatibility, biosafety, and biodegradability of polysaccharide-based hydrogels, used as 3D scaffolds for bone healing. A photocrosslinked composite hydrogel was synthesized under UV irradiation, merging a novel, water-soluble phosphate-functionalized chitosan (CSMAP), prepared with methacrylic anhydride (MA) and phosphonopropionic acid (P), and strontium phosphosilicate (SPS) bioceramic nanoparticles. The CSMAP-SPS hydrogel’s porous network amplified mechanical strength and bioactive ion release. This hydrogel demonstrated superior biomineralization, cytocompatibility with preosteoblast MC3T3-E1 cells, and encouraged osteogenic differentiation and endothelial tube formation, suggesting potential utility in bone regeneration (Chen et al., 2021).

## 8 Others smart polymeric biomaterials for bone regeneration

Several stimuli-responsive biomaterials warrant further exploration, particularly in mechanical, magnetic, and acoustic domains. In the mechanical field, Wolff’s 1892 hypothesis proposed bone’s responsiveness to biophysical stimuli, shedding light on bone and tissue healing as well as the impact of workouts and machine-induced stress on bone and mesenchymal tissue development (Ma et al., 2023b). On the other hand, specific polymers are capable of responding to compression, shear and other mechanical stimuli with network structural change or polymeric degradation, which affect bone implant design and drug delivery. PLA scaffold reinforced with 20% magnesium demonstrated 2.4 times of degradation rate in the presence of 3 MP static compression during a 30-day period, while fluid shear stress greatly increased PLAG degradation (Chu et al., 2017; Chu et al., 2019). The results suggest that carefully chosen stress-responsive polymers may play important roles in bone regeneration. Magnetic-responsive biomaterials also show potential, particularly when pristine superparamagnetic iron oxide nanoparticles (pSPIONs) are incorporated into additively manufactured scaffolds. Such scaffolds, composed of chitosan (CS), poly (vinyl alcohol) (PVA), and hydroxyapatite (HA), exhibit enhanced magnetic properties useful for magnetic hyperthermia and bone regeneration. Notably, the presence of pSPIONs increases cell adherence, proliferation, and ALP expression in human osteosarcoma Saos-2 cells, making these scaffolds a promising choice for bone regenerative applications (Tavares et al., 2023). The synergy of low-intensity pulsed ultrasound and lipid microbubbles with 3D-printed PLGA/TCP scaffolds has also been demonstrated to enhance bone marrow stem cell growth and differentiation, representing a potential strategy for bone regeneration (Jin et al., 2023). Furthermore, titanium-hydroxyapatite and titanium-wollastonite composites exhibit physicochemical and biocompatible properties conducive to future bone implants, underscoring the potential of metal-ceramic composites in bone implant advancements (Shanmuganantha et al., 2022).

## 9 Discussion and conclusion

Smart biopolymers exhibit potential in bone regeneration via innovative scaffold construction, material enhancement, and tailored drug delivery, thereby providing diverse therapeutic avenues. As reviewed before, light-responsive polymers enhance antibacterial effectiveness and bone repair in 3D-printed scaffolds and photothermal treatments. Thermo-responsive materials, conductive polymers, and pH-responsive biomaterials have demonstrated potential in controlled drug release, improved cellular behavior, and combating osteoporosis and infections, respectively. Also, enzyme-responsive polymers and redox signaling pathways targeting materials have shown promise in bone regeneration and infection mitigation. Stimuli-responsive materials have made advances in the mechanical, magnetic, and acoustic domains. Notably, magnetic-responsive biomaterials enhance cell adherence and proliferation, while acoustic-responsive materials stimulate stem cell growth and bone differentiation. The advantages and limitations of smart biopolymers were summarized in Table 1.

**TABLE 1:** Smart polymeric biomaterials: representative references, advantages and limitations.

Type of stimuli	Ref.	Materials	Application	Highlight	Advantages	Limitations
Light- responsive polymeric biomaterials	Zhang et al. (2022)	Shape-memory polyurethane (SMPU)/ Magnesium	Bone scaffold with osteogenic effects	Light weighted and strong, tight contact with bone tissue, robust bone regeneration	1. Non-invasive; 2. Excellent spatial and temporal control; 3. Excellent intensity control; 4. Mild reaction	1. No deep tissue penetration; 2. Less effective in complexed physiological conditions; 3. Possible non- specific tissue reactions to light
	Zeng et al. (2020)	Polydopamine-IR820- daptomycin on titanium implant	Antibacterial; MSC proliferation and differentiation	Antibiotic/photodynamic/ photothermal triple therapy for outstanding antibacterial effects and excellent osseointegration performances		
	Kuang et al. (2021)	PTH, calcium phosphate, PNAm, DHCP-10PIP/d, APS/TEMED	PTH release; Delivery of bone matrix components	Controlled and stable dual mode PTH release; Well-balanced osteoblast and osteoclast activities for in situ micropore formation		
	Wan et al. (2022)	Polydopamine magnesium calcium carbonate hydroxybutyl chitosan hydrogel	Aspirin and BMP-2 release for antibacterial and osteogenic effects	Relief of acute inflammatory reaction and maximized therapeutic effects for bone regeneration		
Thermo- responsive polymeric biomaterials	Duan et al. (2020)	GA, NIPAM, DMAPMA, Montmorillonite	drug carrier for colon delivery	A high-strength galactomannan- based hydrogel with thermal and pH responsiveness	1. Good biocompatibility, biodegradability, and the ability to mimic *in vivo* environments 2. Promotes cell adhesion and proliferation	1. Inherent weak mechanical properties and strong shrinkage of hydrogels can hinder their clinical application 2. Use of chemical agents for cross-linking to improve mechanical properties can increase cytotoxicity
	Bian et al. (2023)	SIM, SrHPO4), β- TCP, PCLA-PEG- PCLA	Injectable composite for bone regeneration in cranial defects	Providing necessary mechanical support and osteoinduction. Enhanced bone regeneration capacity		
	Lei et al. (2022)	ETN, PLGA–PEG– PLGA	Injectable hydrogel system to inhibit wear debris-induced osteolysis in patients undergoing total joint arthroplasty	Effectively neutralizing TNF-α and significantly reducing titanium particles-induced aseptic inflammation and subsequent osteolysis		
	Lv et al. (2023)	GF, BMP-2, MgFe- LDH, CS, PDGF-BB	Construction of a smart injectable thermo-responsive hydrogel for efficient bone regeneration	Promoting angiogenesis and osteogenesis		
Electrically- responsive polymeric biomaterials	Karanth et al. (2023)	PLLA, Fibrinogen	Craniofacial implants	Satisfactory osteoblast-like cell adherence	1. Beneficial for stimulating cellular activities 2. Facilitate bone regeneration through persistent endogenous electrical stimulation	Impact the material's mechanical properties
	Ma et al. (2023a)	PUF, PVDF-HFP, CaP	1. Bone tissue regeneration 2. Treatment of long- term osteoporosis	Promote cell osteogenic differentiation and ectopic bone formation		
	Li et al. (2022)	PMMA, ACNTs	Bone regeneration through electric and magnetic stimulation	incorporating ACNTs into a PMMA matrix, showcasing promise for bone tissue engineering its stimulus-responsive, mechanical, and cytocompatible properties		
	Qiao et al. (2022)	Silicon dioxide electret, PDMS	Electrical stimulation	Exhibits a stable and tunable electrical potential, promotes cellular activity, and enhances osteogenic differentiation		
PH-responsive polymeric biomaterials	Deng et al. (2020)	PEEK, Polydopamine, AgNPs, Apatite	Anti-infection and bone regeneration	3D-printed scaffold with excellent antibacterial and osteogenic effects	Effective in protecting bone structure from acid environments due bacteria and osteoclast activities, especially in combating tooth caries and certain pathogens	1. Sensitivity may be low due to varying *in vivo* pH value; 2. Relatively slow responsive speed; 3. Possible adverse tissue reactions for some polymers
	Chen et al. (2023)	minocycline, PDMAEMA hydrogel, chitosan	Anti-infection and bone regeneration	pH-responsive microfluidic device with preciously controlled drug lease; Long-lasting effects		
	Gan et al. (2015)	BMP-2, Dex, chitosan, MSNs	Osteoblast differentiation and accelerated bone regeneration	Dual-delivery system for two-step drug release for optimized effects		
	George et al. (2022)	OPF, PEG, DOPA	Osteointegration and osteogenesis for implant	Adhesive hydrogel to improve osteointegration with osteogenic effects		
Enzyme- responsive polymeric biomaterials	Ding et al. (2020)	PG, PAH, AgNPs encapsulated MSNs; polydopamine- modified Ti substrates	Bacteria inhibition and bone regeneration	Modified titanium implant with excellent antibacterial effect and significantly improved new bone formation	1. Great biocompatibility, selectivity and efficiency; 2. Fast response and degradation in response to specific enzymes	1. Non-specific targeting for enzymes in the same family; 2. Enzyme dysregulation in certain diseases; 3. Short-lasting activities
	Li et al. (2019)	chitosan membrane containing PPEM	Bacteria inhibition and periodontal tissue repair	Enhanced ALP expression with polyphosphoeste; ALP-responsive membrane for controlled drug delivery; Highly effective in bacteria inhibition		
	Qi et al. (2019)	MPC, bisacryloylated VPLGVRTK peptide, BMP-2	MMP-induced BMP-2 release for bone repair	Effective drug delivery using nanocapsules via malformed blood vessels on fracture sites		
	Yuan et al. (2018)	Vancomycin, dopamine-modified HA; 3,4- dihydroxyhydrocinna mic acid-modified chitosan	Bacteria inhibition and osteointegration	Bacteria-triggered drug lease for minimum side effects; Improved osseointegration		
Redox- responsive polymeric biomaterials	Nilawar et al. (2023)	Ceria polyurethane	Biodegradable multifunctional biomaterials for bone tissue regeneration	Enhancement of polyurethane properties for potential application in bone tissue regeneration	1. Enhances radical- scavenging potential and osteogenic differentiation 2. Supports cell proliferation and differentiation 3. Exhibit high bioactivity and a strong antioxidant capacity	Poor dispersion in the polymer matrix. There's a challenge of ensuring the oxygen supply does not become excessive, as it could disrupt the redox balance, leading to oxidative stress and impeding bone regeneration
	Nilawar and Chatterjee (2022)	PLA, poly(ethylene imine) ceria	Faster bone healing by scavenging reactive oxygen species (ROS)	Enhances their bioactivity for bone tissue regeneration, demonstrating ROS scavenging and antibacterial capabilities		
	Tan et al. (2022)	PgC(3)Mg) , GelMA	Use in the repair of large bone defects	Enhanced osteogenic capability		
	Mac et al. (2022)	RA-MC fibers incorporating nanosilicon (RA- MC/nSi)	Aid in the reconstruction of large bone defects exceeding the natural self-healing capacity of the bone	Guiding cell migration, regulating redox homeostasis, mitigating inflammation, and enhancing osteogenic differentiation		

Despite the considerable potential of smart biopolymers in bone regeneration, obstacles persist. Interactions of biomaterials depend on factors like size, charge, and shape, and the application of responsive polymers remains challenging due to deep tissue penetration limits, mechanical properties variability, and potential induction of oxidative stress. In conclusion, remarkable advances in bone regeneration have been made, but the ideal polymeric materials for this purpose remain to be developed. Their biophysical and biochemical properties should be further exploited to guide material design and fabrication. Additionally, more theoretical and experimental studies are needed to facilitate controllable manipulation to explore their potentials. Furthermore, interdisciplinary collaborations with artificial intelligence (AI) may also foster designs of polymeric biomaterials for bone repair. AI-assisted techniques such as multi stimuli-responsive methodologies and robocasting may enable biomaterial customization with potential breakthroughs in pore shape control and deep tissue penetration to unlock their full potential in bone regeneration.

## Figures and Tables

**Table:** 

**ACNTs**	Activated carbon nanotubes
**ACP**	Amorphous calcium phosphate
**ALP**	Alkaline Phosphatase
**BMP-2**	Bone morphogenetic protein-2
**BNTF**	Ferroelectric BaTiO3 nanofibers
**BP**	Black phosphorous
**BTE**	Bone Tissue Engineering
**CaP**	Calcium phosphate
**Ceria**	Cerium oxide
**CG-NCs**	Cellulose-gelatin (CG) integrated with cerium oxide (nanoceria)
**CHW**	Chitin whisker
**CMCh**	Carboxymethyl chitosan
**CS**	Chitosan
**CSMAP**	Water-soluble phosphate functionalized chitosan
**DAP**	Daptomycin
**Dex**	Dexamethasone
**DHCP**	Poly (dimethylaminoethyl methacrylate-co-2-hydroxyethyl methacrylate)
**DMAPMA**	N-[3-dimethylamino)propyl]methylacrylamide
**DOPA**	Dopamine
**ECM**	Extracellular matrix
**ETN**	Etanercept
**FN**	Fibronectin
**GA**	Galactomannan
**GelMA**	Gelatin methacrylate
**GF**	Growth factor
**HAP**	Hydroxyapatite
**hBMP-4**	Human bone morphogenetic protein-4
**LDH**	Layered double hydroxide
**L-PRF**	Lyophilized platelet-rich fibrin
**MA**	Methacrylic anhydride
**Mg** ^**2+**^	Magnesium ion
**MHBC**	Methacrylated hydroxybutyl chitosan
**MMPs**	Matrix metalloproteinases
**MPC**	2-(methacryloyloxy)ethyl phosphorylcholine
**MSNs**	Mesoporous silica nanoparticles
**n (BMP-2)**	BMP-2 nanocapsules
**NIPAM**	N-isopropylacrylamide
**OHA**	Oxidized hyaluronic acid
**OPF**	Oligo [poly (ethylene glycol) fumarate]
**P**	Phosphonopropionic acid
**P(VDF-TrFE)**	Poly (vinylidene fluoridetrifluoroethylene)
**PAH**	Polyallylamine hydrochloride
**PCL**	Poly (ε-caprolactone)
**PCLA-PEG-PCLA**	Poly (ε-caprolactone-co-D,L-lactide)-poly (ethyleneglycol)-poly (ε-caprolactone-co-D,L-lactide)
**PDGF-BB**	Platelet-derived growth factor-BB
**PDMAEMA**	Poly [2-(dimethylamino) ethyl methacrylate]
**PDMS**	Poly (dimethylsiloxane)
**PEEK**	Polyetheretherketone
**PEI**	Poly (ethylene imine)
**PG**	Poly-l-glutamic acid
**PgC (3)Mg**	Magnesium-seamed C-propylpyrogallol[4]arene
**phBMP-4**	pSTAR-hBMP-4 plasmid
**PLA**	Poly (lactic acid)
**PLA-AP**	Poly (l-lactic acid)-block-aniline pentamer-block-poly (L-lactic acid)
**PLGA/HA**	Poly (lactic-co-glycolic acid)/hydroxyapatite
**PLGA–PEG–PLGA**	Poly (lactide-co-glycolide)-b-poly (ethylene-glycol)-b-poly (lactide-co-glycolide)
**PLLA**	Poly (l-lactic acid)
**PMMA**	Polymethyl methacrylate
**PNAm**	Poly (N-acryloyl glycinamide-co-acrylamide) PNAm
**PPEM**	Polyphosphoester and minocycline hydrochloride
**pSPIONs**	Pristine superparamagnetic iron oxide nanoparticles
**pSTAR**	Plasmid vector
**PTH**	Parathyroid hormone
**PUF**	Polyurethane foam
**PVDF-HFP**	Piezoelectric poly (vinylidene fluoride-co-hexafluoropropylene)
**RA-MC/nSi**	Radially aligned mineralized collagen (RA-MC) fibers incorporating nanosilicon
**ROS**	Reactive oxygen species
**SIM**	Simvastatin
**SMPU**	Shape-memory polyurethane
**SPS, Sr**_**5**_ **(PO**_**4**_ **)2SiO**_**4**_	Strontium phosphosilicate
**SrHPO**_**4**_	Strontium hydrogen phosphate
**TG**	Transglutaminase
**WH**	Whitlockite
**Zn@rGO**	ZnO coating of reduced graphene oxide
**β-TCP**	Beta-tricalcium phosphate
**ε-EPL**	Poly-ε-L-lysine
