# Supplementary material for: The role of smart polymeric biomaterials in bone regeneration: a review
Source: Front Bioeng Biotechnol. 2023 Aug 17;11:1240861. doi: 10.3389/fbioe.2023.1240861 (PMC10469876; doi:10.3389/fbioe.2023.1240861)
Supplement: Supplementary file 1 [file Table1.DOCX]

| **Methods** | **Materials** | **Ref** |
| --- | --- | --- |
| 3D-Printing | Shape memory polyurethane | [32] |
| 3D Printing | PWH and PCL | [54] |
| 3D-Printing | L-PRF-CS-HAP (P-C-H) composite scaffold | [44] |
| 3D-Printing | PLLA | [51] |
| 3D-Printing | PEEK, AgNPs and pDA | [60] |
| 3D-Printing | Nanoceria, ROS and PLA | [71] |
| 3D-Printing | CS, PVA, HA | [80] |
| 3D-Printing | PLGA, TCP | [81] |
| 3D PUF scaffold coated with PVDF-HFP and modified by CaP mineralized coating | PUF, PVDF-HFP and CaP | [52] |
| ACNTs were prepared by a CVD method. ACNTs were added into the solid component of PMMA, the solid and liquid components were mixed and poured into the Teflon mold for polymerization-hardening | PMMA and ACNTs | [55] |
| Adding an hBMP-4 gene fragment into a pSTAR to form the pSTAR-hBMP-4. Combining a triblock copolymer of PLA-AP and PLGA/HA to fabricate the PLGA/HA/PLA-AP/phBMP-4. | PLGA, HA, PLA-AP, phBMP-4 | [58] |
| Ag nanoparticles were pre-encapsulated in MSNs by one-pot method. Then PG and PAH were assembled by LBL assembly technique on MSN-Ag to form LBL@MSN-Ag. | PG, PAH, LBL, MSN-Ag | [64] |
| Asymmetric microfluidic/chitosan device | PDMAEMA hydrogel | [61] |
| BMP-2-functionalized MgFe-LDH nanosheets into CS hydrogels loaded with PDGF-BB | BMP-2, Mg-Fe-LDH, CS, PDGF-BB | [45] |
| Butyraldehyde and Pg were added to methanol followed by HCL. PgC_3_ and MgCl_2_·6H_2_O were dissolved in *N*,*N*-dimethylformamide, then heated overnight. | Pg, PgC_3,_ MgCl_2_·6H_2_O | [73] |
| Chi-c were developed on Ti substrates modified with TiO_2_ nanotube arrays loaded with an antibacterial drug. | Chi-c, HA-c and TiO_2_ | [68] |
| Conjugation of DOPA and the synthetic oligomer OPF | DOPA and OPF | [63] |
| CS membrane loaded with Minocycline hydrochloride (MH) which’s prepared with a PPE crosslinker | CS, MH, PPE | [65] |
| CS was dissolved into acetic acid deionized water then MA was slowly added. EDC and NHS were dissolved into CSMA solution, thus the CSMPA was obtained. CSMPA solution mix with SPS to form CSMAP-SPS hydrogels. | CS, MA, SPS | [76] |
| DHCP hydrogel loaded with PTH | DHCP and PTH | [36] |
| Dissolved calcium chloride in distilled water and adding ammonia solution and PEG | sodium alginate hydrogel, Vitamin C，PEG | [75] |
| Free radical polymerization | Xyloglucan-co-Methacrylic Acid/Hydroxyapatite/SiO_2_ | [15] |
| Free radical polymerization | ARX-g-(Zn@rGO)-HAp | [46] |
| Free radical polymerization | MMPs, BMP-2, MPC and VPLGVRTK | [66] |
| Freeze drying technique | Sodium alginate, hydroxyapatite, and silica with different GO amounts | [4] |
| Freeze drying technique | BC, *β*-G, n-Hap and GO | [10] |
| Freeze drying technique | Arabinoxylan/graphene-oxide/nHAp-NPs/PVA | [13] |
| Freeze drying technique | AAc/GO/nHAp/TiO_2_ | [14] |
| Freeze drying technique | Arabinoxylan-co-AA/HAp/TiO_2_ | [16] |
| Freeze-drying technique | ARX, BG, nHAp, GO and AAAc | [47] |
| Freeze-drying technique | EDC-NHS, C, G and EW | [72] |
| Freeze-drying technique and free radical polymerization | BC-g-(Fe_3_O_4_/GO) | [57] |
| GA was dissolved in deionized water, NIPAM, DMAPMA and MBA were added and stirred evenly. APS and tetramethylethylenediamine was added and the solution were poured into the corresponding mold to form a hydrogel. | GA, NIPAM and DMAPMA | [39] |
| HBC was synthesized by conjugating the ring-opening product of 1,2-butane oxidation onto the chitosan chain | BMP-2 and HBC | [37] |
| HBC were prepared by grafting hydroxyl butyl group on chitosan chain using etherification reaction, then the freeze-dried HBC formed a physical/chemical dual-crosslinking using MHBC and CHW | M/C hydrogel (MHBC/CHW) | [40] |
| Hot press compression and vacuum sintering method | Ti-HAp, CaO_3_Si | [82] |
| In situ co-precipitation method | RA-MC/nSi | [74] |
| Load SIM and SrHPO_4_ andβ-TCP to a thermogel, constituted by PCLA-PEG-PCLA via a thermos-induced sol-gel transition to form an injectable composited scaffold | SIM, Sr, β-TCP, PCLA-PEG-PCLA | [41] |
| MSNs were prepared by a CTAB-templated sol-gel method, and further coated by chitosan via the crosslinking of GPTMS | BMP-2, Dex, MSNs and GPTMS | [62] |
| OHA and ε-EPL were freeze-dried and dissolved into the solution using distilled water as solvent. Then F127 was dissolved into the solution and mix with the OHA and ε-EPL solution. | FHE hydrogel (OHA, ε-EPL and F127) | [43] |
| Polyurethane was synthesized using olive oil-based polyol to impart degradability to the composite | Ceria, PU | [70] |
| SiO_2_ electrets were homogeneously dispersed in the PDMS matrix, and sandwich-like composite membranes were fabricated using a facile layer-by-layer blade-coating method | SiO_2_, PDMS | [56] |
| Solvent exfoliation of bulk BP crystals (MoPhos) then blended into PLGA | BPs and PLGA | [34] |
| SrCl_2_ was added to DCM and the mixture was sonicated, BPs redispersed in PLGA solution in DCM. After sonication, adding DCM containing SrCl_2_ and centrifuged to obtain the BP-SrCl_2_/PLGA | BP-SrCl_2_/PLGA | [38] |
| Synthesized aqueous solutions of thermosensitive copolymers (PLGA-PEG-PLGA) with comparable molecular weight but different LA/GA. | ETN, TNF-alpha antagonist and PLGA-PEG-PLGA | [42] |
| Ti immersed into PDA solution to prepare Ti-PDA, then immersed into IR820 and then DAP | Ti-PDA-IR820-DAP | [35] |
